# Silver nanoparticle as an alternate to antibiotics in cattle semen during cryopreservation

**DOI:** 10.1590/1984-3143-AR2022-0030

**Published:** 2023-11-13

**Authors:** Arushi Kanwar, Meenakshi Virmani, Sant Lal, Kartik Chaudhary, Sandeep Kumar, Ankit Magotra, Anand Kumar Pandey

**Affiliations:** 1 Department of Veterinary Physiology and Biochemistry, Lala Lajpat Rai University of Veterinary and Animal Sciences, Hisar, Haryana, India; 2 Division of Bio and Nano Technology, Guru Jambheshwar University of Science and Technology, Hisar, Haryana, India; 3 Forest Department-Wildlife Wing, Paonta Sahib, Himachal Pradesh, India; 4 Department of Veterinary Gynaecology and Obstetrics, Lala Lajpat Rai University of Veterinary and Animal Sciences, Hisar, Haryana, India; 5 Department of Animal Genetics and Breeding, Lala Lajpat Rai University of Veterinary and Animal Sciences, Hisar, Haryana, India

**Keywords:** CASA, freezing, nanoparticle, silver, sperm

## Abstract

The proposed study was to determine if the silver nanoparticles can be used as potential antimicrobial agents and can replace the use of conventional antibiotics in semen without affecting the motility and fertility of semen. The silver nanoparticles prepared by chemical reduction method were confirmed by determination of the wavelength of surface plasmon resonance peak and further characterized using Zetasizer by determining their size, polydispersity index, and zeta potential. The nanoparticles were assessed for antibacterial activity and their concentration was optimized for use in semen extender for cryopreservation. Cryopreserved semen was further evaluated for seminal parameters, antioxidant parameter, and microbial load. Prepared silver NPs showed a plasmon resonance peak at 417 nm wavelength. NPs were found to possess antibacterial activity and were supplemented in semen extender @ 125 and 250 µg/ml for semen cryopreservation. There was a significant increase in pre and post-freezing motility and other seminal parameters. The microbial load of frozen-thawed semen of control and supplemented groups were well within the permissible limits. Lipid peroxidation levels were reduced in NPs supplemented groups, and reactive oxygen species (ROS) levels were significantly reduced in semen supplemented with 125 µg/ml NPs. Thus it can be conclude that silver NPs can be successfully used as a substitute for antibiotics in cattle bull semen cryopreservation with good antimicrobial activity and no adverse effects on sperm characteristics.

## Introduction

Spermatogenesis involves the formation of functional and mature spermatozoa which are able to fertilize with the egg of a female. Several bull factors, such as health, nutrition, environment, genetic and semen manipulation after collection (eg: cryopreservation) also affect semen quality and its fertility potential (Bungum et al., 2007). Nonviable spermatozoa produce reactive oxygen species (ROS) and decrease antioxidant defence that causes reduced sperm motility, DNA damage and redox imbalance (Schuppe et al., 2008). Bacterial contamination of semen by various pathogenic bacteria such as *Escherichia coli, Staphylococcus aureus, Pseudomonas aeruginosa, Staphylococcus epidermidis, Klebsiella* spp*.,* etc. also deteriorate the quality of semen and affect the life span of sperm, thereby reducing the fertility potential of semen (Pineda and Santander, 2007). Antibiotics are being used to combat bacterial growth. The indiscriminate use of antibiotics is causing increased bacterial resistance to various antibiotics and this multidrug resistance is leading to difficulty in the management of infectious diseases caused by pathogenic bacteria (Maheshwari et al., 2016).

In today’s era of immense antibiotic usage for treatment, disease control, and as growth promoters in livestock, the damaging effects of emerging antimicrobial resistance (AMR) are already manifesting themselves across the world. Multi-drug resistant infections are currently affecting human lives across the globe; however, there are no reliable estimates of the true burden that might be occurring to the livestock and through the food chain to the human species. As antibiotics become less effective, infections such as pneumonia, tuberculosis, blood poisoning, gonorrhoea, and food borne diseases become more difficult, and sometimes impossible to treat. A political declaration issued by the United Nations General Assembly in New York in September 2016 addressed the root causes of antimicrobial resistance across human health, animal health, and agriculture sectors, by starting a joint WHO and Drugs for Neglected Diseases initiative (DNDi) initiative that aims to develop and deliver new treatments by 2023 by improving existing antibiotics and hastening the introduction of new antibiotic drugs. (WHO.in). Thus it becomes essential to develop some alternative novel approaches to prevent antimicrobial resistance.

Nanotechnology is a promising field of research and has a wide application in science and technology, especially for developing new materials. It deals with the particles of smaller size (nanometers), termed as nanoparticles that are stronger, lighter, more durable, and more reactive with improved performance. Nanoparticles due to their unique physio-chemical properties are attracted towards diseased cells, thus allowing earlier detection of disease and its treatment as well as reducing the damage to healthy cells in the body. Iron oxide (Fe_3_O_4_) functionalized with gold and silver has shown promise in biotechnological applications such as protein separation, cancer diagnosis biosensors (Abdelhamid and Wu, 2014). Silver has low cytotoxicity (Biel et al., 2011) and has potent activity as an antiseptic and antimicrobial against various bacteria (Lazar, 2011; Taraszkiewicz et al., 2013). Thus silver nanoparticles (AgNPs), among various nanoparticles, have been considered as potential substitutes to deal with a variety of bacterial pathogens. Silver and gold nanopaticles have also been used to determine the freshness of fruits and vegetables by advanced non destructive method of Raman spectroscopy (Gopal et al., 2016). Modified polyethylene glycols (PEGs) have been reported as potent antibacterial agents for sewer treatment because of their effective cytotoxicity and magnetic property (Abdelhamid et al., 2015). The negative charge on surface of bacteria may be neutralized by silver nanoparticle, which can lead to altered the cell membrane permeability and subsequently cause the cell death (Tang and Zheng, 2018). Silver NPs have a large surface-to-volume ratio (Oves et al., 2018) as well as low cytotoxicity and immunological response (Samuel et al., 2020), thus enabling them to interact with the cell surface of pathogenic bacteria. This damages the cell membranes that lead to structural changes, which makes bacteria more permeable (Lazar, 2011; Periasamy et al., 2012) AgNP enter bacteria and interact with cellular structures and biomolecules such as lipids, proteins, and DNA, inhibiting transcription, translation, protein synthesis, and disrupting cellular functioning (Rinna et al., 2015) and finally causes bacterial cell death (Rai et al., 2014). Moreover, the factors responsible for the antimicrobial activity include the size as well as zeta potential of the nanoparticles. Since bacteria have a negative surface charge, the nanoparticles with positive zeta potential interact with bacteria and promote an electrostatic interaction, possibly leading to the penetration in bacterial membranes. The damaged membranes lead to the production and release of reactive oxygen species (ROS) that have powerful bactericidal action (Wu et al., 2014). It has also been proposed that nanoparticles can release silver ions, which can interact with the thiol groups of many vital enzymes and inactivate them (Prabhu and Poulose, 2012). Silver nanoparticles due to their antibacterial property (Qais et al., 2019) and ROS scavenging action (Falchi et al., 2018) can be used to prevent cryopreservation induced sperm loss.

The use of various NPs in male reproductive biology is summarizes as below in Table 1.

**Table 1:** Summary of use of nanoparticles in reproduction.

**Nanoparticle used**		**Effect**	**Reference**
1	Iron oxide nanoparticles (IONPs)-antiubiquitin antibodies (Abs) complex	Successful depletion of dead/damaged spermatozoa from buffalo (*Bubalusbubalis*) semen	Bisla et al. (2021)
2	Magnetic nanoparticles (MNPs) based sexing in donkeys	Highly effective in selecting X spermatozoa, without affecting several physiological sperm parameters	Domínguez et al. (2018)
3	Magnetic iron oxide nanoparticles (MNPs)	Magnetic tools (MNP) improve fertility in magnetic assisted cell-sorting or MACS as an effective method	Durfey et al. (2017); Farini et al. (2016)
4	Zinc oxide nanoparticles for cryopreservation	Higher percentage of total and progressive motility in frozen-thawed samples in ZnONPs group compared to control. Chromatin damage and malondialdehyde (MDA) level was significantly lower	Isaac et al. (2017)
5	Silver nanoparticles	Effective antimicrobial agent in porcine sperm to replace conventional antibiotics	Pérez‐Duran et al. (2020)
6	Zinc nanoparticles	Increase mitochondrial activity, enhances membrane integrity, reduce lipid peroxidation, improves total antioxidant capacity in bovine spermatozoa	Jahanbin et al. (2021)
7	Cerium oxide (CeO2)	Motility parameters of semen improved after 48 h of incubation. Integrity of plasma membranes of spermatozoa maintained	Falchi et al. (2018)
8	Selenium nano-particles (Se-NPs)	1.0 μg/ml improved post-thaw sperm quality of Holstein bulls, reduced apoptosis, lipid peroxidation and sperm damage that occurs during cryopreservation.	Khalil et al. (2019)
9	Silver-carbon NPs (Ag@C NPs)	Efficient antimicrobial activity (Minimum Inhibitory Concentration: 3.125–12.5 μg/mL) against the *E. Coli, S. aureus, P. aeruginosa* and strong bactericidal effect on *S. aureus,* and *P. aeruginosa* (Minimum Bactericidal Concentration: 3.125 μg/mL), with no detrimental effect on the percentage of sperm motility plasma membrane integrity, acrosome integrity and normal sperm morphology at concentrations of 15 and 30 μg/mL, respectively, after a cold storage of 48 h.	Yousef et al. (2021)
10	Green synthesized gold nanoparticles (GSGNPs, 5; 10ppm/mL)	Increased motility, livability, and membrane integrity post-equilibration, improved in Tris-extended samples in post-equilibrated and post-thawed semen. Viable sperm increased, early apoptotic, apoptotic, and necrotic sperm decreased @10 ppm GSGPNs.	Ismail et al. (2020)

Thus, keeping in view the antibacterial nature of silver, the present study was designed to use silver nanoparticles as a replacement for antibiotics in semen cryopreservation in order to prevent antimicrobial resistance.

## Methods

### Preparation of silver nanoparticles

Silver nanoparticles were prepared by the chemical reduction method followed by (Alghoraibi and Zein, 2017) with little modifications. Briefly, 10 ml of 1mM solution of silver nitrate was mixed with 10 ml of 1.5% PVP (Polyvinylpolypyrrolidone) dropwise that acts as a stabilizing agent and prevents aggregation. The mixture was kept on a stirrer at 800 rpm for 30 minutes. 1ml of freshly prepared 1mM Sodium borohydride was added to the mixture and again stirred for 5-10 minutes at 800 rpm. The appearance of bright yellow color in the solution indicated the formation of silver NPs. Synthesized nanoparticles solution was poured in a petri plate and heated at 70°C. Once the liquid evaporated, powdered nanoparticles were collected with the help of a spatula.

### Characterisation of silver nanoparticles

In order to characterize the prepared nanoparticles, the most important physical property is particle size. Zetasizer (Zetasizer Nano ZS, Malvern Instruments Ltd., UK (Model Micro-P, range 0.05-550 micron) was used to measure the particle size of dispersed nanoparticles in nanometers, using the technique of Dynamic Light Scattering (DLS). Secondly, the zeta potential (ζ) that measures the effective electric charge on the nanoparticle surface as well as the stability of colloidal dispersions, was determined in millivolts, on Zetasizer Nano ZS, using the technique of Electrophoretic Light Scattering (ELS). . Transmission electron microscopy (TEM) using a JEM-2100 HRTEM was done to determine the shape and size of the synthesised nanoparticles. Drop casted TEM samples were placed on a carbon coated copper grid and allowed to dry completely before being placed at a potential of 20 kV. Crystal structure, phase purity and lattice parameters of as-synthesized silver nanoparticles were investigated by measuring X-ray diffraction pattern using Smart Lab Co. Rigaku, Japan

### Evaluation of antibacterial activity of nanoparticles

The antimicrobial activity of nanoparticles was determined by the agar well diffusion method (Valgas et al., 2007) The size of the zone of inhibition produced by the nanoparticles on the culture plate indicated the presence of antimicrobial activity in the sample. Muller Hilton agar (MHA) powder was dissolved in distilled water and autoclaved at 121^o^ C for 15 minutes at 15 psi pressure. The mixture was allowed to cool and was poured on petri plates. The plates were left undisturbed for 30 minutes for the solidification of agar and were incubated at 37º C for 24 hours to make sure that the media and plates were sterile. The agar plate surface was inoculated by spreading and streaking the microbial inoculums [*Escherichia Coli* (MTCC38) and *Staphylococcus aureus* (Fop 171A)] over the entire agar surface. Silver nanoparticle of different concentrations that is 5, 10, 20, and 50 mg/ml were made with double distilled water as solvent having pH 7. Wells of 6 mm diameter were made using a sterile cock borer and 50 µl of silver nanoparticle solution of different concentrations (5, 10, 20, and 50 mg/ml) were added into the wells. Standard antibiotic discs as control for gram positive and gram negative bacteria were penicillin G for *Staphylococcus aureus* and streptomycin for *E. Coli* having concentration of 10 mcg on each disc. The plates were incubated at 37^o^ C for 24 hours in the incubator. Silver nanoparticles diffused in the agar medium formed a concentration gradient which inhibited the growth of the bacterial strain tested and hence caused a zone of inhibition. The zone extended until the concentration of the NP was insufficient to inhibit the growth of the organism. The diameter of the zone of inhibition was measured with the help of verniercalipers to compare antibacterial activity of different concentrations of nanoparticle and to determine if the antibacterial activity of silver NP change with a change in the dose.

### Freezing of semen supplemented with silver nanoparticles

#### Extender preparation

TRIS-Egg yolk-Glycerol extender was prepared by the Standard CSS (Certified Semen Services) protocol which is a 2-step method in which 30.28gm Tris, 16.75 gm citric acid, 12.00 gm D-Fructose in 1 litre of DDW were used. Egg yolk was added @ 20% and glycerol @ 7% to the volume of buffer (Baracaldo et al., 2006).

#### Semen collection and processing

Semen ejaculates were collected from three mature bulls with the help of a sterilized artificial vagina at weekly intervals after approval from Research Ethics Committee (numbered 1669/GO/ABC/12/CPCSEA). Semen samples were assessed for total volume per ejaculate, total sperm count, and motile sperm count. Sperm motility was assessed under a phase-contrast microscope equipped with a warm stage (37^0^C) at 200X magnification and a total of 20 ejaculates having ≥70% sperm motility were used for cryopreservation. Semen was diluted with an extender to make the sperm concentration 80 million/ml so as to make the final concentration of 20 million spermatozoa/straw which is optimum for artificial insemination in bovines with cryo preserved semen (Mohanty et al., 2018).The diluted semen was divided into three aliquots: one aliquot (group C) was supplemented with antibiotics (Penicillin @ 10 Lakh IU/liter and Streptomycin @ 1gm/liter) and the other two aliquots (Group 1 and 2) were supplemented with 125 and 250 µg/ml silver nanoparticles and the extended semen was loaded into 0.25 ml mini-straws (IMV, France) and equilibrated at 4^0^C in the cold cabinet for 4 h followed by cryopreservation with liquid nitrogen (LN_2_).

#### Cryopreservation of semen

Cryopreservation of sperm is a sequential process of reduction in temperature, dehydration of the cell, freezing and storage (Ugur et al., 2019). Semen sample was filled in 0.25 ml French straws using automatic straw filling machine. The straws were equilibrated at 4°C in cold cabinet for 4 hours and then transferred to biological freezer in which straws were frozen in vapour of liquid nitrogen. The rate of freezing in biological freezer was -19°C/min and the temperature reached -141°C in 7 minutes. The straws were then stored in liquid nitrogen.

### Evaluation of semen

#### Estimation of sperm kinetics and motility by CASA

Two semen straws from each group were thawed in a water bath at 37^0^C for 30 sec. The thawed sample of the straws was transferred to 1.5 ml tubes that were kept in a dry bath at 37^0^C and assessed for sperm kinetics and motility, as well as morphological anomalies using the computer assisted sperm analyzer (Computer-assisted sperm analyzer (CASA) system (IVOS II^TM^ Clinical, Hamilton-Thorne Biosciences, Beverly, MA, USA). Each sample was loaded in one chamber of the eight chambered Leja slide (depth 20 µm) and a minimum of 5 optical fields were selected from each chamber.

The CASA set-up was as follows: Elongation Max (70%), Elongation Min (5%), Head Brightness Min. (114), Head Size Max (50 µm^2^), Head Size Min (11 µm^2^), Tail Brightness Min. (81), Tail Min Brightness Auto Offset (10), Progressive STR (50%), Progressive VAP (37 µm/s), Min Tail Length (4 µm), Stage Temperature (38ºC), Frame Capture Speed (60 Hz) and Frames Count (30).

The motion characteristics that were recorded: Total motility (TM, %), progressive motility (PM, %), average path velocity (VAP, μm/s), straight linear velocity (VSL, μm/s), distance average path (DAP, μm), curvilinear velocity (VCL, μm/s), average lateral head displacement (ALH, μm), beat cross frequency (BCF, Hz), straightness (STR, %), distance curvilinear (DCL, μm), distance straight line (DSL, μm), motile concentration (million/ml), linearity (LIN, %), wobble (WOB) and distal midpiece reflex (DMR %) of the spermatozoa.

#### Estimation of sperm viability

The viability of sperm was estimated by the eosin-nigrosin staining method (Campbell et al., 1960). Briefly, the frozen-thawed semen was mixed with eosin-nigrosin stain, previously warmed at 37°C and a drop of this mixture was placed on a pre-warmed clean grease-free glass slide. A thin smear was prepared, air-dried and the slides were examined under a microscope (1000X), first starting with low magnification (400X) in order to get an overview of the stained spermatozoa. Live spermatozoa did not stain while dead spermatozoa stained pink-red against blue-black background, partially stained spermatozoa counted as dead. Approximately 200 sperms from different fields in a zig-zag manner were counted to calculate the percent live dead sperm.

#### Estimation of acrosome integrity

Giemsa stain was used to determine the percent intact acrosomes as per the method described by (Watson and Martin, 1972). Stock Giemsa stain was prepared by mixing 1gm Giemsa stain, 66 ml methanol, and 60 ml glycerol. This solution was kept at 37ºC, mixed once after every 24 hours for seven days, then filtered and stored at 5ºC. The solution was brought to room temperature (22-25ºC) at the time of use. Working Giemsa stain was prepared fresh by adding 3 ml Giemsa stock solution to 2 ml Sorenson buffer and made the volume 50 ml with distilled water.

Extended semen was used to prepare a smear on a clean, sterilized glass slide that was dried on warm stage at 37ºC for 2-3 min. The smear was fixed in neutral formalin saline solution for 15 min, after which it was rinsed with running tap water. Staining of prepared smear was done by keeping the fixed smear in working Giemsa stain solution for 40 min. The change in acrosome was viewed and estimated using a phase-contrast microscope under oil immersion (100X) lens.

### Evaluation of antioxidant parameters

#### Assay of lipid peroxidation

Malondialdehyde (MDA) is an end product of lipid peroxidation. The method used for malonaldehyde determination was based on the principle that the reaction of MDA with thiobarbituric acid (TBA) yielded a pink-colored complex in an acidic medium. The absorbance was measured at 532 nm wavelength (Ohkawa et al., 1979). Briefly, 0.1 ml sample was mixed with 0.2 ml of 8.1% SDS solution, and 1.5 ml of 20% acetic acid solution was added. the pH of the solution was adjusted to 3.5. 1.5 ml of 0.8% aqueous solution of TBA was added and the final volume was made 4.0 ml with distilled water. The reaction mixture was heated in the water bath at 95ºC for 60 min and then cooled immediately by keeping it in ice-cold water. 1.0 ml distilled water and a 5.0 ml mixture of n-butanol and pyridine (15:1) were added. The test tubes were vigorously shaken and then centrifuged at 4000 rpm for 10 min. The organic layer was separated and the absorbance of the organic layer was taken at 532 nm.

#### Assay of Reactive Oxygen Species (ROS)

The level of ROS production in the samples was assessed by the method used by (Falchi et al.*,* 2018). 25μl of each sample was diluted in 1ml PBS containing 10μM 2′, 7’ dichlorofluorescein diacetate (H2DCFDA) and incubated in the dark for 30 minutes at 38 °C. After incubation, samples were centrifuged at 4229 rpm for 3 minutes to separate the supernatant which was gently discarded and the resultant pellet was again suspended in 500 μL of 2% paraformaldehyde. This mixture was left undisturbed at 4 °C for 1 h. Following fixation, samples were again centrifuged at 4229 rpm for 3 min after which supernatant was removed and 300 μL PBS was added to it. Samples were then stored in the dark at 4°C till flow cytometric analysis. A blue laser (488 nm) was used for excitation purposes and was further detected using the FITC channel. A total of 10,000 events was examined for each sample. The doublets and clumps were excluded for data accuracy using polygon gate and only the main population was gated (Auto Line Segment) under the histogram. The gated spermatozoa were classified as ROS-positive or ROS-negative. All the data collected was analyzed using CytExpert software (v.2.3)

#### Estimation of microbial load of semen

The microbial load of semen was estimated by using the standard plate count (SPC) method (Shukla, 2011) 200μl of sample from frozen-thawed semen was evenly spread over Standard Plate Count agar with a help of a sterile spreader. The plates were incubated at 37°C for 24 h. The number of colonies formed on the plate was counted.

### Statistical analysis

The data were analyzed using the SPSS (Version 23) software package. Sperm quality parameters before and after mixing with the nanoparticles were analyzed using a One-way analysis of variance. The difference among means was tested by Duncan’s multiple range tests. Significant differences were considered when P < 0.05. The values for all parameters were given as mean ± standard error.

## Results

### Preparation of silver nanoparticles

**Figure 1:**
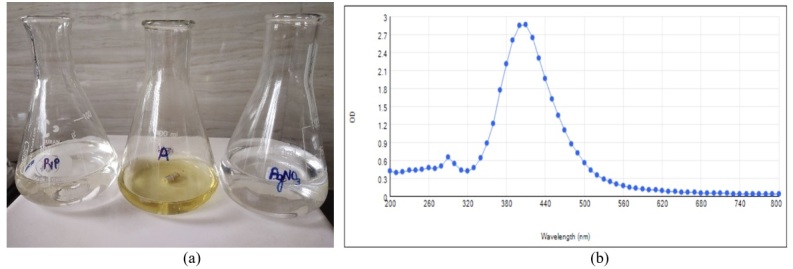
(a) Chemicals used in AgNO3 preparation along with yellow color solution that confirmed NP formation; (b) UV-Vis spectrum for AgNPs.

Silver nanoparticles were prepared using silver nitrate solution, PVP, and sodium borohydride. The appearance of yellow color in the resulting solution indicated the synthesis of silver NPs (Figure 1a) and a surface plasmon resonance peak was observed. Spectra of silver colloids contained a strong plasmon band at 417 nm wavelength. This confirmed the reduction of silver ions (Ag^+^) to Ag° in the aqueous phase (Figure 1b).

### Characterization of silver nanoparticles

**Figure 2:**
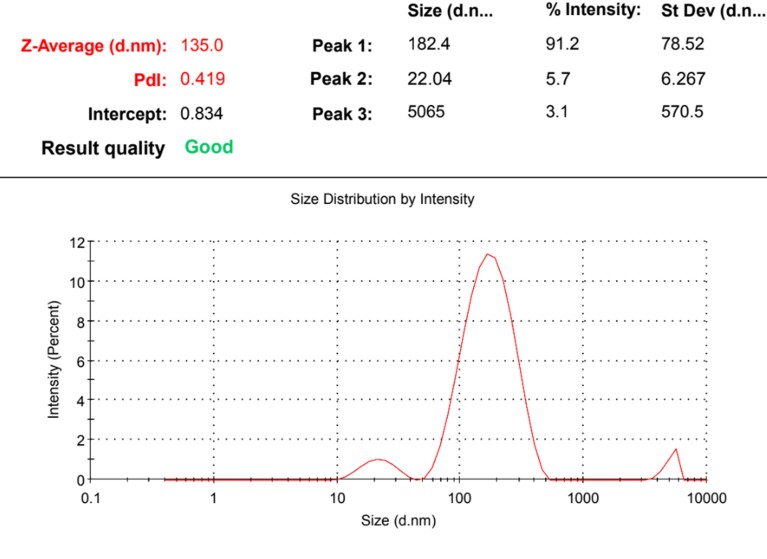
Graph showing Z-average and Pdi of prepared NPs in Zetasizer.

Characterization was done by Zetasizer Nano ZS and z-average, Pdi (Polydispersity Index), and zeta potential was measured for different batches of NPs prepared. The mean z-average for the prepared NPs was 130.25 ± 6.92d.nm, ranging from 112 to 145d.nm. PdI indicated whether NPs formed are monodisperse (particles of the same size) or polydisperse (particles of different sizes). The normal range of PdI is 0-1 and values more than 0.1 indicated polydisperse particles. Zeta potential indicated the net charge on NP formed and the stability of formed NPs in colloidal solution mixture. Its value ranges between -60 to +60 mV and values between -30 to +30 mV are considered to be more stable. NPs were formed in four batches and the values of different parameters are given in Table 2. Figure 2 shows a graph showing Z-average and Pdi of one of the prepared batches of NPs in Zetasizer.

**Table 2:** Average particle size, PdI and Zeta potential of different batches of silver NPs prepared by chemical method.

**Batch Number**	**Z- Average (d.nm)**	**PdI (Poly dispersity index)**	**Zeta potential (mV)**
1	129	0.411	-2.74
2	112	0.561	-2.93
3	135	0.419	-2.16
4	145	0.303	-2.04
**Mean ± SE**	**130.25 ± 6.92**	**0.42 ± 0.05**	**-2.47 ± 0.30**

**Figure 3:**
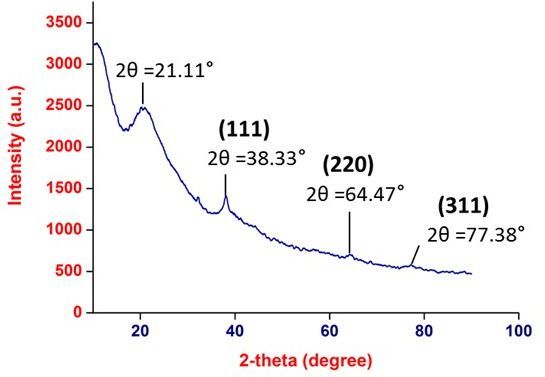
XRD analysis of AgNP.

The obtained diffractogram in XRD imaging (Figure 3) indicated the peaks at 38.33°, 64.47° and 77.38°, which attributed to (111),(220), and (311) lattice planes respectively. The obtained crystal planes clearly indexed as face centred cubic (FCC) structure, which also signified that the as-synthesized Ag nanoparticles were crystalline in nature. The broad peak in the amorphous region at 21.11° is of PVP, as we have synthesized PVP cappedsilver nanoparticles. Due to amorphous nature of PVP, it masked the peak at 64.46° and 77.38°, which may be the reason for small peaks. The diffraction pattern obtained was regarded as consistent with International Centre for Diffraction Data (ICDD).

**Figure 4:**
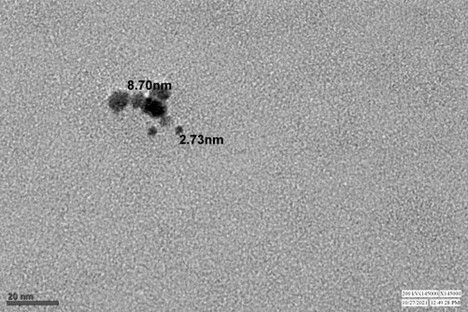
TEM micrograph of AgNP.

The distribution, shape and size of the silver nanoparticles were analysed by TEM analysis. It was seen that the silver nanoparticles were non-uniformly distributed and spherical in shape with size ranging from 2.73 nm to 8.70 nm (Figure 4).

### Evaluation of antibacterial activity of silver nanoparticles

**Figure 5:**
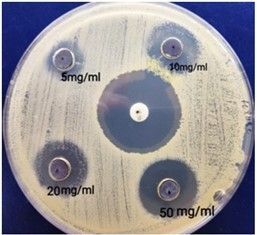
Zone of Inhibition obtained by different concentrations of AgNP and standard antibiotics on *E. Coli*.

**Figure 6:**
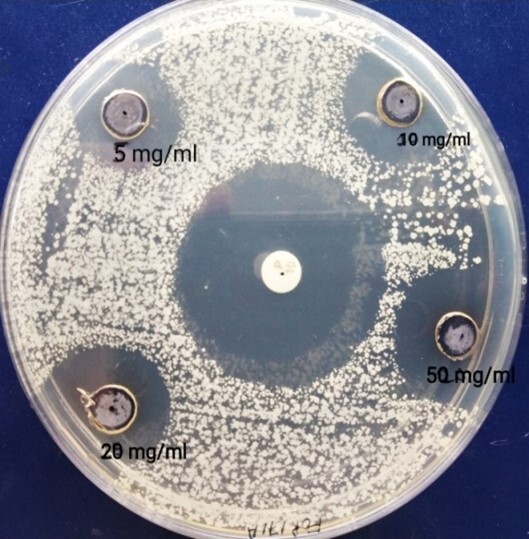
Zone of Inhibition obtained by different concentrations of AgNP and standard antibiotics on *S. aureus*.

The antimicrobial activity of NPs was assessed by the agar well diffusion method and different concentrations of silver NPs were used against *Escherichia Coli* and S*taphylococcus aureus*. The zone of inhibition was measured (in mm) after 24 h of incubation at 37º C to determine the antibacterial activity (Table 3). The size of the zone of inhibition by silver NPs increased from 8.67 ± 1.52 to 10.33 ± 0.61mm on *E. coli* culture (Figure 5) and from 10.33 ±1.05 to 13.00 ± 0.51mm on *S. aureus* culture (Figure 6) as the concentration of NPs increased from 5 to 50 mg/ml. The presence of a modest but well-established clean zone surrounding the AgNPs well is significant evidence of antibacterial activity. Also, antibacterial effect of silver nanoparticle increased in a dose dependent manner.

**Table 3:** Size of zone of inhibition (mm) on bacterial culture using different concentrations of nanoparticles.

**Concentration of NP (mg/ml)**	**Zone in *E. coli* culture**	**Zone in *S. aureus* culture**
5	8.67 ± 1.52	10.33 ±1.05
10	9.83 ± 0.48	12.67 ± 1.14
20	9.67 ± 0.71	12.83 ± 0.54
50	10.33 ± 0.61	13.0 ± 0.51
Antibiotic	32.33 ± 0.71	30.17 ± 1.30

### Effect of supplementation of silver nanoparticles on sperm kinetics and motility

The percent motile and progressive motile spermatozoa were observed to be comparable in control, 1 and 2 groups, however, the increase in progressive motile percent was significant in group 1 after freezing. There was non-significant variation in motile concentration of sperms and other sperm abnormalities (bent tail, coiled tail, and distal droplet) showed non-significant variation post freezing among control, 1 and 2 groups (Table 4).

**Table 4:** Effect of silver nanoparticles on the motility and abnormality parameters before and after freezing.

**Parameter**		**Control**	**Group 1** **(NP- 125 µg/ml)**	**Group 2** **(NP- 250 µg/ml)**	**p value**
Motile (%)	Before Freezing	61.3 ± 2.81	66.25 ± 2.70	63.7 ± 2.04	0.392
	After Freezing	36.95 ± 1.81	41.83 ± 1.42	39 ± 1.31	0.085
Progressive motile (%)	Before Freezing	44.4 ± 2.68	48.2 ± 3.06	46.45 ± 1.94	0.589
	After Freezing	28.35^b^ ± 1.84	35.99^a^ ± 1.40	32.1^ab^ ± 1.77	0.009
Motile Conc. (Million/ml)	Before Freezing	17.15 ± 2.69	17.75 ± 3.06	18.5 ± 2.60	0.943
	After Freezing	7.68 ± 0.92	10.94 ± 1.02	8.51 ± 1.21	0.087
Bent Tail Million/ml	Before Freezing	0.00	0.00	0.00	-
	After Freezing	0.18 ± 0.08	0.39 ± 0.23	0.35 ± 0.14	0.06
Coiled Tail	Before Freezing	0.00	0.00	0.00	-
	After Freezing	0.0055 ± 0.005	0.007 ± 0.005	0.01 ± 0.005	0.830
Distal droplet	Before Freezing	0.00	0.00	0.00	-
	After Freezing	1.13 ± 0.14	1.43 ± 0.19	1.20 ± 0.28	0.579
DMR %	Before Freezing	1.15 ± 0.18	1.05 ± 0.15	1.20 ± 0.19	0.826
	After Freezing	1.14 ± 0.14	1.43 ± 0.19	1.20 ± 0.28	0.595

Mean values with different superscript in a row differ significantly (p<0.05).

DAP and DSL did not show any variation before freezing but increased significantly after freezing in supplemented groups. DCL showed non-significant variation among control, 1 and 2 groups before and after freezing. ALH decreased significantly after supplementation before and after freezing. There was a non-significant variation in VAP, VCL, and VSL among the three groups, however, VAP and VSL showed a slight increase in supplemented groups after freezing. BCF was significantly higher in the control group before freezing but there was a non-significant change in BCF values after freezing among the groups. The relative kinematic parameters *viz.,* WOB, LIN, and STR increased significantly in supplemented groups both before and after freezing (Table 5). The values of actual kinematic parameters after supplementation of silver NPs before and after freezing are presented in Table 6. The effect of supplementation of silver NPs on before-freezing and after-freezing abnormality and motility parameters is shown in Tables 4, 5 and 6.

**Table 5:** Effect of silver nanoparticles on the relative kinematic parameters in semen before and after freezing.

**Parameter**		**Control**	**Group 1** **(125 µg/ml)**	**Group 2** **(250 µg/ml)**	**p value**
WOB %	Before Freezing	59.6^b^ ± 1.17	63.2^a^ ± 1.03	62.5^a^ ± 0.70	0.03
	After Freezing	63.7^b^ ± 0.92	68.25^a^ ± 1.36	69.25^a^ ± 1.20	0.003
LIN %	Before Freezing	51.45^b^ ± 1.53	56.85^a^ ± 1.42	56.2^a^ ± 0.80	0.009
	After Freezing	56.2^b^ ± 1.22	62.25^a^ ± 1.50	63.55^a^ ± 1.22	0.00
STR %	Before Freezing	84.25^b^ ± 0.98	88.15^a^ ± 1.01	88.3^a^ ± 0.53	0.002
	After Freezing	86.85^b^ ± 0.85	89.80^a^ ± 0.54	90.45^a^ ± 0.53	0.001

Mean values with different superscript in a row differ significantly (p<0.05).

**Table 6:** Effect of silver nanoparticles on the actual kinematic parameters in semen before and after freezing.

**Parameter**		**Control**	**Group 1** **(NP- 125 µg/ml)**	**Group 2** **(NP- 250 µg/ml)**	**p value**
DAP μm	Before Freezing	21.20 ± 0.85	21.85 ± 0.83	21.65 ± 0.87	0.859
	After Freezing	29.88^b^ ± 1.05	33.12^ab^ ± 1.64	38.05^a^ ± 2.40	0.008
DCL μm	Before Freezing	36.65 ± 1.50	35.1 ± 1.30	35.5 ± 1.41	0.723
	After Freezing	51.15± 2.01	49.71 ± 2.70	56.60 ± 3.93	0.237
DSL μm	Before Freezing	17.9 ± 0.82	19.35 ± 0.87	19.15 ± 0.82	0.423
	After Freezing	27.5 ± 1.29^b^	29.98^ab^ ± 1.58	34.8^a^ ± 2.28	0.017
ALH μm	Before Freezing	6.60^a^ ± 0.24	5.60^b^ ± 0.25	6.00^ab^ ± 0.14	0.008
	After Freezing	6.97^a^ ± 0.31	6.14^b^ ± 0.26	5.92^b^ ± 0.20	0.017
VAP μm/s	Before Freezing	81.95 ± 2.11	78.75 ± 3.23	81.55 ± 2.50	0.651
	After Freezing	97.85 ± 4.83	99.76 ± 3.34	104.45 ± 5.06	0.566
VCL μm/s	Before Freezing	139.25 ± 4.60	125.9 ± 4.93	131.45 ± 3.81	0.701
	After Freezing	158.05± 7.23	149.00 ± 6.14	154.85 ± 7.93	0.663
VSL μm/s	Before Freezing	70.45 ± 1.87	70.4 ± 3.10	73.05 ± 2.36	0.115
	After Freezing	86.2 ± 4.58	90.84 ± 3.22	96 ± 4.80	0.274
BCF Hz	Before Freezing	25.8^b^ ± 0.44	27.60^a^ ± 0.47	27.25^a^ ± 0.48	0.019
	After Freezing	27.23 ± 0.71	27.72 ± 0.82	29.02 ±0.88	0.279

Mean values with different superscript in a row differ significantly (p<0.05).

### Effect of supplementation of silver nanoparticles on sperm viability, sperms with intact acrosome, antioxidant parameters and microbial load of semen

Frozen thawed semen supplemented with different concentrations of NPs was checked for various morphological parameters including viability (%), abnormality (%), acrosome integrity (%), and various antioxidant parameters. Table 7 data shows that there was no significant variation between the control group and supplemented groups (groups 1 and 2) in the case of the viability of sperm in fresh (before freezing) and frozen-thawed (after freezing) semen. There was a significant increase in percent spermatozoa with intact acrosome in frozen-thawed semen in group 1 in comparison to the control group and group 2.

**Table 7:** Effect of supplementation of silver nanoparticles on sperm livability, acrosome integrity and antioxidant parameters of semen.

**Parameter**		**Control**	**Group 1** **(125 µg/ml)**	**Group 2** **(250 µg/ml)**	**P value**
Livability (%)	Before Freezing	71.58 ± 1.16	71.27 ± 1.98	71.11 ± 1.66	0.823
	After Freezing	58.83 ± 2.70	61.50 ± 2.04	59.33 ± 2.05	0.751
Acrosome Integrity(%)	Before Freezing	97.75 ± 0.46	98.5 ± 0.53	97.87 ± 0.64	0 .08
	After Freezing	62.83^b^ ± 1.35	71.50^a^ ± 1.47	64.00^b^ ± 1.87	0.005
Lipid peroxidation (nmol MDA/g)	After Freezing	609.78±77.71	534.27 ±108.04	473.42 ± 67.67	0.591
Reactive Oxygen Species (ROS) (%)	After Freezing	3.82^b^± 0.92	2.02^c^± 0022	9.68^a^ ± 0.93	0.00001

For lipid peroxidation assay, it was recorded that with the increase in the concentration of silver NPs in the semen extender, the level of lipid peroxidation was significantly reduced as compared to control group in frozen-thawed semen. ROS levels showed a significant difference between the control and supplemented groups, ROS concentration increased in group 2 significantly, however, the value of ROS showed a significant decrease at lower concentration of silver NPs supplementation in group 1 as compared to control values.

### Effect of supplementation of silver nanoparticles on microbial load of semen

The results of the present study showed no or very less microbial load, that is, very much within the permissible limit in control, group 1 and group 2. The standards for acceptable colony forming units (CFUs) in processed semen are upto 5000 per ml as per OIE norms (DHADF, 2014). The result of the microbial load is given below in Table 8.

**Table 8:** Effect of supplementation of silver nanoparticles on microbial load of semen (Colony Forming Unit count).

**Control**		**Group 1** **(125 µg/ml)**	**Group 2** **(250 µg/ml)**
Mean ±SE	0.83 ± 0.48	1.33 ± 0.49	8.83 ± 8.03

## Discussion

The UV–Vis spectroscopic appearance of a single and strong absorption peak at 417 nm confirmed the indicated successful NP formation using silver nitrate, sodium borohydride, and PVP [poly (N-vinyl-2pyrrolidone). Average hydrodynamic diameter of 130.25 nm and Pdi of 0.42 indicates monodisperse nature of NPs. Zeta potential of -2.47 indicate that NP’s formed were stable in colloidal solution. The XRD imaging clearly indexed crystalline nature ofas-synthesized AgNP.TEM imaging of NPs showed successful preparation with non-homologouspolydispersic distribution of sheet like nanoparticles. Similar studies used for the preparation of silver NPs reported the appearance of single and strong absorption peak (surface plasmon resonance) ranging between 397- 408 nm (Alghoraibi and Zein, 2017) and 415–425 nm (Fayaz et al., 2010; Rahman et al., 2018) in UV-Vis spectra and as the concentration of silver increases in the solution, the absorption band became sharper.

Measuring the zone of inhibition is a rapid and inexpensive method for determining the susceptibility of a particular antigen to the bactericidal agent (Yu, 2007). Zone of inhibition for both gram positive and gram negative bacteria was found to increase in a dose dependant manner thus establishing the antimicrobial activity of AgNO_3_ prepared. Pérez-Duran et al. (2020) also reported that silver nanoparticles (AgNPs) have a biocidal effect in bacteria and fungi microorganisms with no toxicity to mammalian cells. SilverNPs possess strong antimicrobial activity against *Stapylococcus warneri* (Dong et al., 2017), *Escherichia coli*, *Staphylococcus aureus* and *Pseudomonas aeruginosa* with minimum inhibitory concentration (MIC: 43 3.125 - 12.5 g/mL) of Ag@C NPs against the tested strains, as well as a strong bactericidal effect on *S. aureus* and *P. aeruginosa* (MBC: 3.125 g/mL), with no detrimental effect on sperm motility (Yousef et al., 2021). Some other studies (Birla et al., 2009; Ingle et al., 2008; Gade et al., 2010; Raheman et al., 2011) also confirmed the antibacterial potential of silver NPs.

Antibacterial effect of silver nanoparticles increased in a dose dependent manner in the present study. Although zone of inhibition in case of NPs is smaller as compared to standard antibiotics, that might be due to low proclivity of silver NPs for the agar (Ong, 2022). Because of the nanoparticle’s multi-targeting methods, the antibacterial action of AgNP is generally unaffected by antibiotic resistance mechanisms (Rai et al., 2012). The nanoparticle could also help with the existing problem of controlling chronic bacterial infections, which are frequently accompanied with the colonisation of naturally durable biofilms (Radzig et al., 2013). The presence of a modest, well-established clean zone surrounding the AgNPs well is significant evidence of antibacterial activity that increased in a dose dependent manner without causing any harm to sperm cells as proven by morphological evaluations.

The motility parameters in the present investigation showed a significant increase at a lower dose @125 µg/ml of NP supplementation. Post-thaw seminal parameters *viz*., intact acrosome and percent abnormal sperms were found to be improved in semen samples supplemented with nanoparticles as compared to the control group. Lipid peroxidation and ROS levels showed a decline in nanoparticle supplemented groups in the present study. The microbial load of semen supplemented with NPs was negligible and well under the permissible limits, that is <5000 cfu/ml (as per OIE guidelines) in the present study. Silver ions and silver-based compounds are well known for high toxicity to microorganisms since the beginning of the 1990s (Akçan et al., 2020). The findings of our study are in accordance with those of other workers (Yousef et al., 2021) who reported no detrimental effect on acrosome integrity, plasma membrane integrity, and normal morphology of sperm when Ag@C NP (AgNPs embedded in carbon) were supplemented in the semen at the concentrations of 15 and 30 µg/ml but concentrations ≥60 μg/ml showed damaging effects on bull sperm parameters (Pérez-Duran et al., 2020). Supplementation of Se-NPs at the rate of 0.5 and 1.0 μg/ml in extended bull semen improved the ultrastructure of the sperm, sperm characteristics, and antioxidant activity in freeze-thawed semen (Khalil et al., 2019). Cerium oxide (CeO_2_) NPs act as ROS scavengers due to their ability to store oxygen and when supplemented to ram semen, it maintained ram sperm cell viability during cooling, improved motility parameters, and increased sperm velocity after 48 hours and up to 96 hours of incubation (Falchi et al., 2018).

## Conclusion

The advantageous effects of silver might have been potentiated due to the reduction of silver into a nano-sized particle, which increased the bioavailability and reduced the unnecessary and undesired liberation of toxic concentrations of silver ion. Silver nanoparticles can be successfully used as a viable substitute to antibiotics in cattle bull semen cryopreservation without affecting the motility and other seminal parameters. Moreover, these nanoparticles showed strong antioxidant and antibacterial activity. Antibiotic-free antimicrobial formulations for semen cryopreservation will be helpful in preventing the occurrence of bacterial multidrug resistance. However, further investigation on *in vitro* and *in vivo* validation of treated semen is required to be done to study the effect on fertilizing ability of spermatozoa.

## Abbreviations

NP - nanoparticle, ROS - reactive oxygen species, ART - assisted reproductive technology, AgNP - silver nanoparticle, PVP - poly vinyl pyrrolidone, PEG - polyethylene glycol, TM - total motility, PM - progressive motility, VSL - straight linear velocity, VAP - average path velocity, VCL - curvilinear velocity, ALH - average lateral head displacement, BCF - beat cross frequency, STR - straightness, DAP - distance average path, DCL - distance curvilinear, DSL - distance straight line, LIN - linearity, WOB – wobble, DMR - distal mid piece reflex.
